# BERT-based tourism named entity recognition: making use of social media for travel recommendations

**DOI:** 10.7717/peerj-cs.1731

**Published:** 2023-12-21

**Authors:** Dhomas Hatta Fudholi, Annisa Zahra, Septia Rani, Sheila Nurul Huda, Irving Vitra Paputungan, Zainudin Zukhri

**Affiliations:** Department of Informatics, Universitas Islam Indonesia, Yogyakarta, Indonesia

**Keywords:** BERT, Named entity recognition, Traveling, Recommendation

## Abstract

**Background:**

Social media has become a massive encyclopedia of almost anything due to its content richness. People tell stories, write comments and feedback, and share knowledge through social media. The information available on social media enables ‘clueless’ travelers to get quick travel recommendations in the tourism sector. Through a simple query, such as typing ‘places to visit in Bali’, travelers can get many blog articles to help them decide which places of interest to visit. However, doing this reading task without a helper can be overwhelming.

**Methods:**

To overcome this problem, we developed Bidirectional Encoder Representations from Transformers (BERT)-based tourism named entity recognition system, which is used to highlight tourist destination places in the query result. BERT is a state-of-the-art machine learning framework for natural language processing that can give a decent performance in various settings and cases. Our developed tourism named entity recognition (NER) model specifies three different tourist destinations: heritage, natural, and purposefully built (man-made or artificial). The dataset is taken from various tourism-related community articles and posts.

**Results:**

The model achieved an average F1-score of 0.80 and has been implemented into a traveling destination recommendation system. By using this system, travelers can get quick recommendations based on the popularity of places visited in the query frame.

**Discussion:**

Based on the survey that we conducted to target respondents who have never visited and have no or limited knowledge about tourist attractions in some example cities, their average interest level from the recommendation results is higher than four on a scale of 1 to 5. Thus, it can be considered a good recommendation. Furthermore, the NER model performance is comparable to another related research.

## Introduction

For some people, traveling is a thrilling experience. It allows them to try new things like visiting new places, meeting new people, and learning about the area’s culture. According to World Tourism Organization (UNWTO) figures, the number of international tourists arriving in the United States grew from 2017 to 2019 (UNWTO, 2020). Due to the COVID-19 epidemic, it declined substantially in 2020. On the other hand, global tourism increased by 4% in 2021 compared to the previous year. International visitor visits could increase by 30% to 78% in 2022 compared to 2021 and only return to 2019 levels in 2024 or later, according to UNWTO expert forecasts (UNWTO, 2022).

People usually plan which destinations they wish to see before going on vacation. Various Internet or social media sources serve as their primary information and inspiration while planning a vacation. Social media has altered the way people interact and connect, including travel, during the last decade. Friends, travel bloggers, and influencers use Instagram, Facebook, Twitter, and other social media to share their vacation photographs and tales. According to a survey, 87 percent of millennials used Facebook to plan their vacations, and more than half utilized Pinterest or Twitter (Condor Ferries, 2023). According to another study conducted by Schofields, over 40% of millennials aged 18–33 consider “Instagrammability” while choosing a vacation destination (Schofield, 2017)). Such social media messages have a definite impact on a traveler’s decision to go where they want to go next. However, making a decision can be difficult and time-consuming, especially when visiting new regions or traveling alone. Furthermore, the abundance of sources frequently leads to new issues. It is understandable that carefully analyzing all sources to gather useful information can be overwhelming.

Named entity recognition (NER) is a method that is believed to solve these issues. NER will extract and classify information from the text available on any Internet source or social media platform based on specified tourism entities or categories to deliver attraction recommendations. NER assigns labels (classes) or semantic categories to words or phrases found in a text (Mansouri, Affendey & Mamat, 2008). This type of recognition helps determine what users look for when they search (Eftimov, Seljak & Korošec, 2017).

In NER task, there are three basic approaches: lexicon-based, rule-based, and machine learning approaches. Maximum entropy, support vector machines (SVM), hidden Markov model (HMM), conditional random fields (CRF), decision tree, and the hybrid method are the examples of these approaches (Kaur & Gupta, 2020). Besides CRF that is well-known to be the model in NER task (Agrawal et al., 2022), recurrent neural network (RNN), including its successor, long short-term memory (LSTM), becomes the pioneer of DL architecture that handles sequence modeling, as in sentences. However, a new approach that can be used in NER modeling task, bidirectional encoder representations from transformers (BERT). BERT is a self-attention-based language model representation pre-trained on several language modeling tasks utilizing raw unlabeled texts (Taher, Hoseini & Shamsfard, 2020). BERT can blend the left and right context that enables the pretraining of a bidirectional deep transformer. It removes unidirectional limitation. The fine-tuned, pre-trained BERT models might perform well without external resources or feature extraction, and it is provenly found to be effective (Agrawal et al., 2022). In Agrawal et al. (2022), BERT-based model outperformed other models such as CRF and the combination of bidirectional long short-term memory (Bi-LSTM) with the CRF model.

 

NER has been researched in a variety of domains. The BiLSTM-CRF model is utilized in the biomedical arena to recognize drug names in biomedical publications (Zeng et al., 2017). The input vector for this investigation was created by concatenating word embedding and character-level embedding. In the biological area, (Boudjellal et al., 2021) used an Arabic dataset to do NER and found that employing a pre-trained monolingual BERT case model rather than a multilingual BERT case model yielded the best results. Sobhana, Mitra & Ghosh (2010) uses the IITKGP-GEOCORP dataset with the CRF approach to work on NER in the geological domain. The IITKGP-GEOCORP is a geology-related data set compiled from various publications and journals published in India. A NER model was created in the archeology area to annotate text collections with archeological named entities (Brandsen, Verberne & Lambers, 2022). An approximately 10 GB of Mongolian dataset used in Cheng et al. (2020) contained various Mongolian news and Mongolian Tourism obtained from various websites. Bouabdallaoui et al. (2021) built a Moroccan dataset that was collected from TripAdvisor Moroccan forum which contained 46,120 topic threads. The Korean and English tourism datasets in Jwa & Jwa (2022) were constructed using tourism information data from the VisitJeju web of the Jeju Tourism Organization, internet websites, and smart tourism information system. Their dataset contains 20,768 words for Korean NER learning data, while the English NER learning data has 12,024 words. NER research has also been done in a variety of languages, including Portuguese (Souza, Nogueira & Lotufo, 2019), Turkish (Güneş & Tantuǧ, 2018), and Bengali (Drovo et al., 2019). In Labusch, Neudecker & Zellhöfer (2022), German datasets were used. They used BERT to create the model. Their findings show that a properly trained BERT model may perform well in various of contexts and even deliver state-of-the-art performance in many circumstances without requiring substantial fine-tuning and optimization. Hakala & Pyysalo (2019) performed NER on the Spanish dataset. They used a CRF-based baseline technique and multilingual BERT to complete the PharmaCoNER task on Spanish biological named entity recognition. For NER in Persian, Taher, Hoseini & Shamsfard (2020) employed the BERT model. For identifying named entities in the Chinese language, Zhuang et al. (2021) used a bidirectional gated recurrent unit with CRF. To produce high-quality word vectors, they also use a pre-trained BERT model. Gao, Zheng & Zhao (2021) combined BERT with BiLSTM and CRF and used a Chinese dataset. The BERT-BiLSTM-CRF model outperforms the Word2vec-BiLSTM-CRF model, according to the results. Their best model can correctly recognize various entity categories.

NER has been used in various works in the tourism domain (Cheng et al., 2020; Vijayakrishna & Sobha, 2008; Saputro, Kusumawardani & Fauziati, 2016; Hu, Nuo & Tang, 2019; Chantrapornchai & Tunsakul, 2021; Xue et al., 2019). CRF was used in Vijayakrishna & Sobha (2008) to perform tourism named entity recognition using Tamil dataset which obtained an F1-score of 80.44%. A semi-supervised NER was developed in Saputro, Kusumawardani & Fauziati (2016) to find new tourist attractions. However, another two-stage idea (YATSI), naive Bayes classifier (NBC), and K-nearest neighbor (KNN) are the methodologies used in that research and it obtained an average F1-score of 69%. Using BERT and other techniques, (Hu, Nuo & Tang, 2019) extracts the Chinese Tourism field attribute, such as location, time, and Chinese name; Chantrapornchai & Tunsakul (2021) extracts location, accommodation, and facility from TripAdvisor, Traveloka, and Hotels.com; Xue et al. (2019) extracts person, location, organization, and time from Trip.com and Mafengwo app. Recently, BERT has been used in Cheng et al. (2020) to create a corpus for Mongolian tourism and obtained more than 80% score. BERT looks promising for NER.

A BERT-based NER model is proposed in this article to support tourism destination recommendations from social media posts. As there is still no similar research that we found, this research becomes one of the pioneers in using BERT-based NER to be the core of recommendation engine. This research is also conducted to see BERT’s performance for NER modeling on only English tourism dataset. In the Materials & Methods section, the materials and methods used in the study are described. Results and discussion are presented in the Results and Discussion. Finally, the Conclusions section concludes the paper.

## Materials & Methods

**Figure 1:**
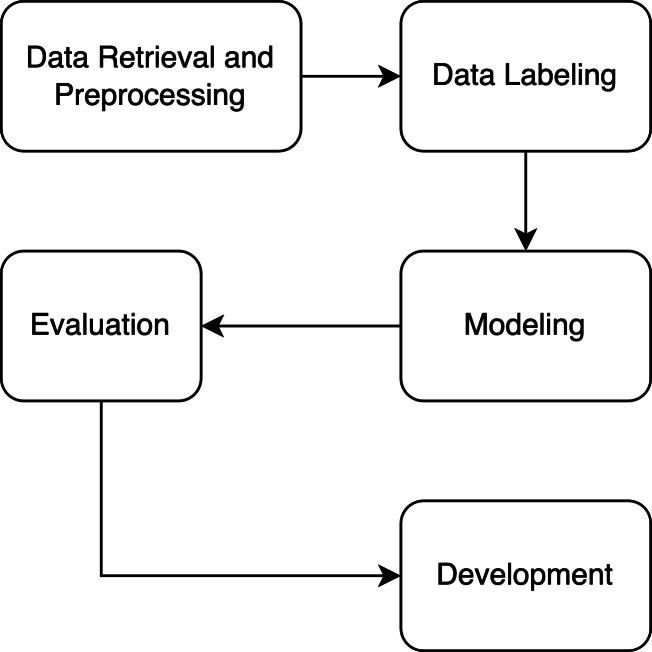
The research methodology of the study.

The aim of our resaerch is to develop a recommendation engine in tourism domain that is based on BERT-based NER. Figure 1 shows the research flow. The research starts with data retrieval and preprocessing step. The required and related datasets are collected and is preprocessed to produce clean dataset. A clean dataset is then labeled with tourism categories. In the modeling step, BERT based model architecture is used. The developed model will be evaluated to see its performance. Finally, the best model build will be implemented into a proof-of-concept application.

### Data retrieval and preprocessing

We gathered the data for this study by crawling the web for numerous English travel articles, such as worldofwanderlust.com, touropia.com, tourmyindia.com, vaticantour.com, myguidezimbabwe.com, daytours4u.com, and Traveloka.com. The search was initially conducted by entering several keywords alternately into the Google search engine, such as “top cities for tourists”, “best places to visit”, “top world heritage sites”, “world heritage list”, “best destinations for nature lovers”, and “best natural tourist attractions”. The data obtained up to that point was preprocessed, which included the removal of emoticons and URLs, as well as tokenization. We opted to remove the emoticons and URLs because they did not contribute anything to the process of recognizing a named entity. On the other hand, tokenization is breaking down sentences into their constituent elements, known as tokens. Tokenization is done at the word token level, where the analysis and inference process occur. We collected 183,507 tokens from 92 articles with 8,500 sentences and 17,137 different words.

### Data labeling

Swarbrooke & Page (2002) proposed tourist attraction categories, namely natural, man-made/artificial purpose-built and non-tourist purpose-built, and special events. Included in the first category are tourist attractions in the form of natural products such as beaches, mountains, and forests. The second category includes tourist attractions explicitly built to attract tourists, such as parks, museums, and galleries. The third category contains tourist attractions whose development goals are not to attract tourists, for example, historical buildings or places such as monuments, temples, and castles. Cathedrals and churches, which are places of worship, also fall into the third category. The last category is festivals and other events, such as sporting events.

Such labels are partially adopted in our study. Natural is used to classify places with rivers, waterfalls, mountains, beaches, and other places that offer natural scenery that is open to the public. Man-made purpose-built and man-made non-tourist purpose-built categories are combined into a single category, called Purpose. It covers purposefully built (man-made) tourist attractions, such as amusement parks and museums. An additional category is Heritage. Tourist attractions that are classified as Heritage are those that have been around for many years. They are historical, cultural, ancient, or representatives of a culture, such as castles, mosques, ruins, and cathedrals. In contrast to Swarbrooke (2002), we do not specifically have a Special event category due to the nature of such an event that is not always available.

We employed the IOB tagging format in this work, with B-label indicating a token at the start of a noun phrase, an I-label indicating a token within the current noun phrase, and O-label indicating otherwise. B-prefix correlates to the first word of a tourist attraction’s name in this study, whereas I-prefix corresponds to the second through the last word of a tourist attraction’s name. The labeling process is done manually by the author. The total number of tokens for each label is shown in Table 1. We have reasonably balanced entities in each of the three tourist attraction categories. Due to the nature of expressing descriptive articles, the mentioned tourist attraction entity in the dataset has a small portion compared to other non-tourist attraction tokens.

**Table 1:** Number of tokens.

**Label**	**Number of tokens**
O	171,728
B-NATURAL	1,789
I-NATURAL	1,853
B-HERITAGE	1,401
I-HERITAGE	2,051
B-PURPOSE	1,811
I-PURPOSE	2,874

### Modeling

In this study, we used a model that had been pre-trained utilizing BERT to perform the NER task. It was introduced in Devlin et al. (2018) as a technique for pre-training deep bidirectional representations from the unlabeled text. The pre-training was accomplished by simultaneously conditioning all model layers on the right and left contexts.

As BERT depends on the tokenization at the word piece level, we decided to initially perform tokenization at that level before putting the data into the previous trained version of the BERT model. For instance, the word ‘Prehistoric’, which has the label ‘I-PURPOSE’, is tokenized to ‘Pre’, ‘##his’, ‘##tor’, and ‘##ic’. The label for each of these word pieces will be ‘I-PURPOSE’. After the tokenization process is done to all sentences, we add the specialized BERT tokens (‘CLS’ and ‘SEP’). Then, based on the maximum length, we padded or shortened the tokens, and for the next phase, we built the attention mask in addition to the labels using the dictionary that had been defined before this stage. A training dataset and a testing dataset were created from the original dataset used in this work. We adopted an 80:20 split, with 80 percent of the data going toward training and 20 percent testing. We used holdout method for the splitting mechanism.

To select the best model, we applied hyperparameter tuning to the model architecture during the modeling process. Different learning rates, epochs, and batch sizes were tested. We also used two distinct BERT models, BERT-base-cased and BERT-base-uncased, to test our hypothesis. We modify one type of hyperparameter at a time during the experiment while the other hyperparameters are frozen. The base hyperparameter that we used is as follows: learning rate = 0.00001, epoch = 3, training batch size = 4, testing batch size = 2, model = BERT-base-cased. On top of that, Adam optimizer is used. The hyperparameter optimization performed on the testing set.

### Evaluation

After completing the training procedure, we used the testing dataset to evaluate our model’s performance. The F1-score is used to assess its performance. Because most tokens in the NER dataset are labeled as O, which implies that the token is not a named entity, F1-score is a better choice than accuracy (Seok et al., 2016). The F1-score is the harmonic mean of precision and recall, as shown by Eq. (1). The F1-score ranges from 1 to 0, such that 1 being the highest and 0 being the lowest.

(1) \begin{eqnarray*}{F}_{1}=2 \frac{precision\cdot recall}{precision+recall} .\end{eqnarray*}

In addition to the F1-score, a survey on potential users is conducted as part of the evaluation process. The target audience for this survey is ‘clueless’ visitors or those who have little or no information about the city and the tourist attractions they plan to visit. This survey aims to see if the system’s recommendation meets the user’s needs, in other words, how appealing the tourist attractions recommendation is to the user. A 1 to 5 rating scale is used in the survey to capture user interest in the suggested outcome. A rating of 1 indicates that the recommendation is not interesting, while a rating of 5 indicates that the recommendation is very interesting. In the questionnaire, we choose four cities as an example: Kyoto, London, Lisbon, and Colorado. The average user interests for each city is then used to determine the survey results.

### Development

As a proof of concept in delivering the recommendations, a web-based application is built using the best model. The application accepts user query that asks for the tourist destination location that has never been visited. The program will list tourist places in the specified three destination categories based on information acquired from community articles on the web.

## Results

Finding the best hyperparameter setup that results in the best model in terms of F1-score is the first step in the modeling process. Table 2 shows the final hyperparameter using BERT-based-case weightage. The BERT-base-cased model has a linear layer on top of the output of the hidden states, and this layer functions as a token classification layer. These hyperparameters resulting the best model with average F1-score of 0.80. Hyperparameters in Table 2 are chosen through experimenting with different values of each type of hyperparameter. We tried 0.0001 and 0.000001 as the other values in terms of learning rates. By doing so, the F1-score is degraded to 0.61 and 0.29, respectively. Raising the epoch from 2 to 5 does not give significant differences. Hence, we picked 3 as the value of the epoch number. In the batch size sections, we tried to double up the values of the training batch size to 8, 16, and 32, and the testing batch size to 4, 8, and 16. The F1-score result degrades to 0.78, 0.75, and 0.66. Finally, using the BERT-base-uncased model also slightly reduced the F1-score to 0.78.

**Table 2:** Hyperparameter used.

**Hyperparameter**	**Value**
Training batch size	4
Testing batch size	2
Epoch	3
Optimizer	Adam
Learning rate	0.00001

Table 3 shows the performance metrics score for all categories, including precision, recall, and F1-score. Heritage and Natural have a slightly better F1-score than Purpose because they tend to have similar patterns, such as the words that are often used in naming tourist attractions, for example both “Lake Tahoe” and “Lake Toba” used the word “Lake” in their names. So that the model can more easily detect these tourist attractions. Meanwhile, there are more word variations for tourist attraction that fall into the Purpose category. The variances could confound the model and prevent it from making accurate predictions. We also tried to predict new named entities in new input sentences based on the trained model. The prediction result is shown in Table 4. We can see from the table that our model managed to predict all tokens correctly.

**Table 3:** Performance metrics score for each tourist attraction categories.

	**Precision**	**Recall**	**F1-score**	**Support**
HERITAGE	0.81	0.81	0.81	521
NATURAL	0.78	0.85	0.81	796
PURPOSE	0.74	0.80	0.77	722

**Table 4:** Example of prediction result for new sentence.

**Token**	**Predicted label**
Grand	B-NATURAL
Lake	I-NATURAL
is	O
just	O
before	O
the	O
western	O
entrance	O
to	O
Rocky	B-PURPOSE
Mountain	I-PURPOSE
National	I-PURPOSE
Park	I-PURPOSE
.	O

**Figure 2:**
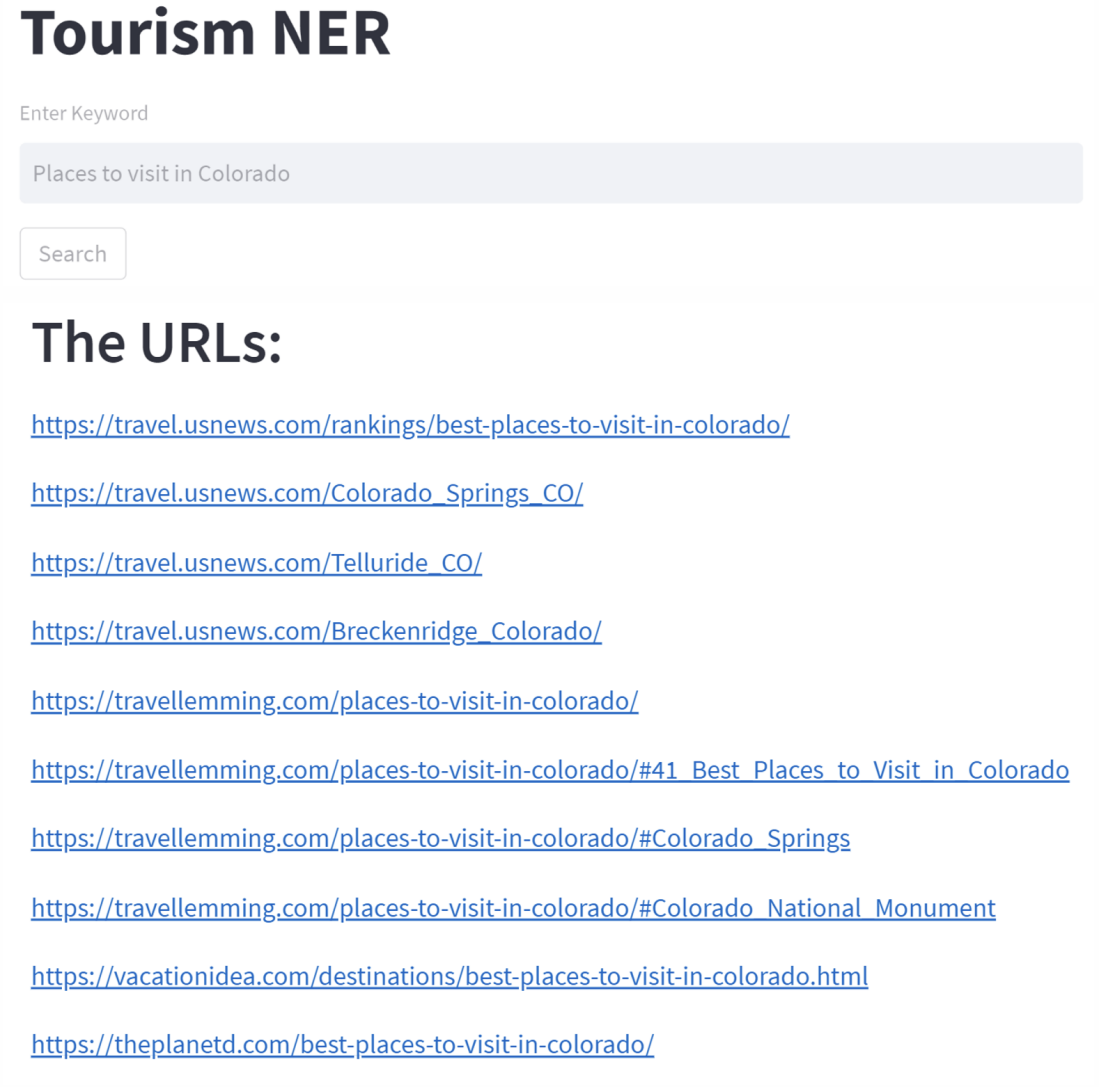
The recommendation application with query sample and result.

We also built a simple web-based application to implement the NER model for extracting tourist attractions from several articles. This web interface is useful for people to use the model that has been built so that they can get tourist recommendations in cities that are not well recognized by users (this is what we define as clueless travelers). As shown in Fig. 2, we tried “Places to visit in Colorado” as the input keyword. The URLs found, which will then be scraped, is also shown in Fig. 2.

We have created a web-based application to use the NER model to extract tourist attractions from various of articles. This web interface allows people to utilize the model created to acquire tourist recommendations in cities that are not well known by users (this is what we define as clueless travelers). We attempted “Places to visit in Colorado” as an input keyword, as shown in Fig. 2. Figure 2 also shows the URLs that hold tourist destination information to be collected later.

**Figure 3:**
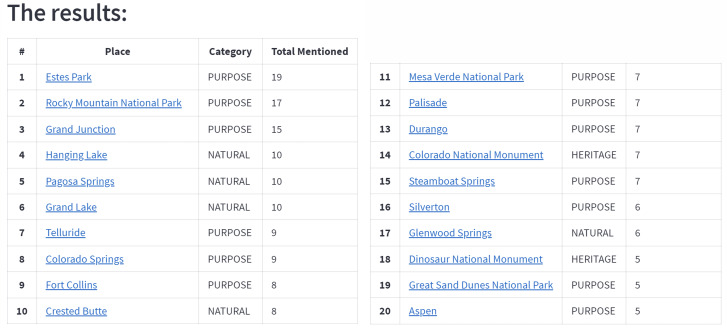
The example of detected tourist attraction in the recommendation application.

The contents of the articles in the URLs were scraped automatically after the URLs were retrieved from Google Search Engine. We can use this method to search travel blogs and reviews as sources. Each word with the entity labels Heritage, Natural, or Purpose will be recorded. The number of occurrences in the article sources is then used to rank the terms. The tourist attractions located in the article sources are presented to the user as the suggestion output after being ranked. The recommendation results for the keyword “Places to visit in Colorado” are given in Fig. 3.

We are not considering whether the review is good or negative in this study. On the other hand, given the keywords selected, good review outcomes are more likely than negative review results. In order to evaluate the suggested results, we ran a survey utilizing a questionnaire with target respondents who had never been and had little or limited information about tourist sites in specific example locations, such as London, Kyoto, Colorado, and Lisbon. These criteria are set to portray the situation and behavior of clueless travelers.

The survey was completed by 40 people ranging in age from 17 to 54. They never went to all cities listed as examples: London, Kyoto, Colorado, and Lisbon. On a scale of 1 to 5, the respondent judged how good is the recommendation result. Table 5 shows the average level of user interest in the sample cities’ recommended tourist attractions. With the average interest level higher than 4 on a scale of 1 to 5, the output from NER in those source articles can be considered a good recommendation.

**Table 5:** Evaluation result from survey.

City	Average of users’ interest
Kyoto	4.18
London	4.01
Colorado	4.14
Lisbon	4.08

## Discussion

As a technique to label a phrase inside a sentence, NER can be utilized in this research to give a tourist attraction recommendation. The recommendation gathered from this approach is a general recommendation that has not considered the user’s personality and preferences. Thus, this kind of recommendation is still suitable for initial ideas of the most attractive places, especially for travelers with no or limited initial knowledge of the city they want to visit. Users do not have to search and read many articles and blogs to gather a list of tourist attraction recommendations.

We have developed a NER model in tourism to detect tourism destinations from any English articles and implemented the model as recommendation engine. The recommendation result is proven to be a good recommendation from the survey conducted with the average interest level higher than 4 in a scale of 1 to 5. However, our approach has some limitations. This study has not yet considered whether the article sources have positive or negative reviews of the tourist destinations mentioned in them. On the other hand, this can be further exploited as an advantage by using positive or negative keywords to gather the desired results. Another limitation is that some article sources may write similar tourist location names in different writing. For example, when using the keyword ‘Places to visit in Lisbon’, the system gave several recommendations, two of them are Jerónimos Monastery and Jeronimos Monastery. Both are treated as two different recommendations, even though the two phrases refer to the same tourist destination. The situation happens because both ways of writing (Jeronimos Monastery/Jerónimos Monastery) are commonly used in the article sources. Our further research will try to detect the similarity in the tourist destination names to avoid such redundancy.

Despite these limitations, the recommendations from our approach are pretty suitable for clueless tourists as our target audience. Our previous research (Rani et al., 2021) only targeted specific users by considering their personalities and preferences. The results from this research complement our previous research to give more elaborate recommendations for tourists. Furthermore, comparing our current BERT-based NER model to our previous study and experiments in developing NER model using BiLSTM-CRF (Zahra, Hidayatullah & Rani, 2022), our BERT-based model is proven to deliver better performance (80% in F1-score) rather than the BiLSTM-CRF-based model (75.25% in F1-score).

If we focus on the NER model itself, study (Cheng et al., 2020; Bouabdallaoui et al., 2021; Jwa & Jwa, 2022; Chantrapornchai & Tunsakul, 2021) has the same purpose as ours in developing the NER model for the tourism domain using BERT-based architecture. Table 6 shows the previous studies and highlights four key points of the studies: datasets, entities, methods, and performance. In the view of datasets, most studies use tourism-related websites and applications to get the data, including ours. While our study is more global in terms of the scope of tourism datasets, other studies specifically focus on tourism in: Mongolian (Cheng et al., 2020), Moroccan (Bouabdallaoui et al., 2021), Korea (Jwa & Jwa, 2022), and Thailand (Chantrapornchai & Tunsakul, 2021). The number of the dataset cannot be compared directly to each other, studies (Cheng et al., 2020) uses the number of sentences, (Bouabdallaoui et al., 2021) uses the number of topic threads, (Jwa & Jwa, 2022) and (Chantrapornchai & Tunsakul, 2021) use the number words/keywords. Our dataset has 8,500 sentences which is half the number of sentences compared to the dataset in Cheng et al. (2020). The target entities to be extracted by the developed NER model are mostly tourism-related destinations or places, even though the label might be different. Study (Cheng et al., 2020) has scenic and cultural categories, (Bouabdallaoui et al., 2021) has gastronomy and religion category, and our study has specific heritage, natural, and purposes categories. BERT has become the base architecture model in the studies, including ours. However, study (Bouabdallaoui et al., 2021) added more BERT-based models such as RoBERTa, and (Jwa & Jwa, 2022) uses KoBERT. Finally, in terms of performance, the F1-score results on all the studies vary. We cannot compare it apple to apple since the dataset and the category in are different. However, we can see that the BERT-based model for tourism NER studies averagely has F1-score ranging from about 70 to 90 percent, where our NER model has 80% as the score.

**Table 6:** Previous studies of BERT-based NER in tourism domain.

**Study**	**Dataset**	**Entities**	**Method**	**Performance (F1-Score)**
Cheng et al. (2020)	Mongolian news and tourism websites (16,000 sentences)	Person, scenic, cultural, organization, spesific field	CRF, BiLSTM-CRF, BERT	78.55%, 80.08%, 82.09%
Bouabdallaoui et al. (2021)	Moroccan TripAdvisor forum (46,120 topic thread)	Regions, hotels, restaurants, shopping places, gastronomy, religion, outfits, transports and touristic guides	BERT, RoBERTa, XML-RoBERTa	70.5%, 69.44%, 68.53%
Jwa & Jwa (2022)	Smart tourism app, visitJeju web, and web search (20,768 words in Korean and 12,024 words in English)	Tourist destination, accomodation, restaurants and cafes	KoBERT-CRF	94%
Chantrapornchai & Tunsakul (2021)	Two different dataset for 8 different provinces in Thailand: 1. Tripadvisor, Traveloka, Hotels.com (58,828 keywords) 2. Tripadvisor website (29,619 keywords after cleaning)	Two different NER tasks: 1. Location, organization, and facility 2. Restaurant, hotels, shopping and tourism	BERT	1. 38%–91% (default tokenization) 2. 77,9% - 98,8%

## Conclusions

Social media and community information on the web in the tourism industry allows clueless travelers to acquire instant vacation recommendations. Travelers can find various blog entries that might assist them in picking which locations of interest to visit during their vacation by typing a basic query and getting automatic recommendations rather than manually DOIng reading tasks, which can be time-consuming.

We created BERT-based tourism named entity recognition model that highlights tourist destinations in one query search results to address this challenge. Our tourism NER model distinguishes three types of tourist destinations: heritage, natural, and purposely constructed (man-made). The data was compiled from various community articles and postings about tourism. We picked the best model from our experiments through the hyperparameter tuning phase. The model has been integrated into a travel location recommendation system with an average F1-score of 0.80. Travelers can get rapid recommendations using this approach based on the popularity of destinations visited in the query context.

To evaluate the recommendation system, we ran a survey to target respondents who have never been and have little or limited information about tourist sites in selected sample cities. The average interest level from the suggestion results is higher than 4 on a scale of 1 to 5. As a result, it is a good recommendation. In addition, the NER model’s performance is equivalent to that of other related studies. Considering future development of the recommendation system, we will use the model and the recommendation result to form a systematic itinerary recommendation for the user.

## Supplemental Information

10.7717/peerj-cs.1731/supp-1 Supplemental Information 1 Code and data used in the research

## Additional Information and Declarations

### Data Availability

The following information was supplied regarding data availability: The modeling code, implementation code, and data are available in the Supplemental Files.
